# Correlates of Recent Declines of Rodents in Northern and Southern Australia: Habitat Structure Is Critical

**DOI:** 10.1371/journal.pone.0130626

**Published:** 2015-06-25

**Authors:** Michael J. Lawes, Diana O. Fisher, Chris N. Johnson, Simon P. Blomberg, Anke S. K. Frank, Susanne A. Fritz, Hamish McCallum, Jeremy VanDerWal, Brett N. Abbott, Sarah Legge, Mike Letnic, Colette R. Thomas, Nikki Thurgate, Alaric Fisher, Iain J. Gordon, Alex Kutt

**Affiliations:** 1 Research Institute for the Environment and Livelihoods, Charles Darwin University, Darwin, Northern Territory, Australia; 2 School of Biological Sciences, The University of Queensland, Brisbane, Queensland, Australia; 3 School of Biological Sciences, University of Tasmania, Private Bag 55, Hobart, Tasmania 7001, Australia; 4 Biodiversity and Climate Research Centre (BiK-F) & Senckenberg Gesellschaft für Naturforschung, Frankfurt, Germany; 5 School of Environment, Griffith University, Nathan Campus, Sydney, Queensland, Australia; 6 Centre for Climate Change and Tropical Biology, School of Marine and Tropical Biology, James Cook University, Townsville, Queensland, Australia; 7 CSIRO—Land and Water—Australian Tropical Sciences Precinct, PMB PO, Aitkenvale, Queensland, Australia; 8 Flora and Fauna Division, Northern Territory Department of Land Resource Management, PO Box 496, Darwin, Northern Territory, Australia; 9 Australian Wildlife Conservancy, PO Box 8070, Subiaco East, Perth, Western Australia, Australia; 10 National Environmental Research Program Northern Australia Hub, Charles Darwin University, Darwin, Northern TerritoryT, Australia; 11 School of Biological, Earth and Environmental Sciences, University of New South Wales, Sydney, New South Wales, Australia; 12 TropWATER, James Cook University, Townsville, Queensland, Australia; 13 School of Earth and Environmental Sciences, Terrestrial Ecosystems Research Network, University of Adelaide, Adelaide, South Australia, Australia; 14 James Hutton Institute, Invergowrie Dundee, Scotland, United Kingdom; 15 ARCUE, School of BioSciences, University of Melbourne, Melbourne, Victoria, Australia; University of Sydney, AUSTRALIA

## Abstract

Australia has experienced dramatic declines and extinctions of its native rodent species over the last 200 years, particularly in southern Australia. In the tropical savanna of northern Australia significant declines have occurred only in recent decades. The later onset of these declines suggests that the causes may differ from earlier declines in the south. We examine potential regional effects (northern versus southern Australia) on biological and ecological correlates of range decline in Australian rodents. We demonstrate that rodent declines have been greater in the south than in the tropical north, are strongly influenced by phylogeny, and are consistently greater for species inhabiting relatively open or sparsely vegetated habitat. Unlike in marsupials, where some species have much larger body size than rodents, body mass was not an important predictor of decline in rodents. All Australian rodent species are within the prey-size range of cats (throughout the continent) and red foxes (in the south). Contrary to the hypothesis that mammal declines are related directly to ecosystem productivity (annual rainfall), our results are consistent with the hypothesis that disturbances such as fire and grazing, which occur in non-rainforest habitats and remove cover used by rodents for shelter, nesting and foraging, increase predation risk. We agree with calls to introduce conservation management that limits the size and intensity of fires, increases fire patchiness and reduces grazing impacts at ecological scales appropriate for rodents. Controlling feral predators, even creating predator-free reserves in relatively sparsely-vegetated habitats, is urgently required to ensure the survival of rodent species, particularly in northern Australia where declines are not yet as severe as those in the south.

## Introduction

The global mammal fauna is declining rapidly [1]. In Australia, many rodent species have gone extinct (10 species) or declined significantly in distribution and abundance (18 species) since European settlement (in 1788), particularly in the semi-arid and arid regions of central and southern Australia (Eremaean bioprovince) [2] and the Mediterranean zone of southern Australia [3, 4]. Although extant rodent species have continued to decline in southern and central Australia [4], until recently, mammals in the northern tropics of Australia appeared stable [5], presumably because some of the threats that operate in the southern regions are absent from the tropics. However, in recent decades, there has been a rapid decline in a suite of mammals of relatively small body mass, including rodents, in tropical northern Australia [6–9]. Identifying the causes of this decline, and whether there are common mechanisms of mammal decline operating in north and south Australia is a high conservation priority [10].

Rodents worldwide are under-represented in conservation efforts [11]. Yet, in modern times at least 56 species of rodents have become extinct worldwide or are presumed extinct (30% of extinct species), and 30% of all currently threatened mammals are rodents [12]. In some parts of the world, such as the Mexican, Caribbean and the Galapagos Islands, diverse radiations of endemic rodents have been extirpated [13, 14]. The endemic rodents of Australia, which once comprised ~30% of the non-flying mammal fauna of the continent, suffered severe declines in the nineteenth and early twentieth centuries, along with many marsupials [4, 10]. In Australia the conilurine rodents comprise 49 species (of 62 rodent species in Australia) and seven endemic genera [15]. This group warrants special conservation attention because it has undergone exceptional declines: 35 species are in decline and eight species on continental Australia are extinct, representing ~4% of global rodent declines and extinctions [4, 16]. Eighty-eight percent (n = 28 species) of conilurine rodent species in southern Australia have declined to some extent, and 37% (n = 7 species) have declined in northern Australia. The decline of this group in Australia is one of the worst proportional fauna losses anywhere, comparable to the loss of Singapore forest endemics [17], Lake Victoria cichlids [18], Guam birds [19], neo-tropical cloud forest frogs [20] and French Polynesian Partulid snails [21].

There is little obvious landscape modification in northern Australia, such as widespread land-clearing, that could explain regional scale small-mammal declines. Several possible causes have been suggested, including changed climate and weather patterns, changed fire regimes (particularly, increase in frequency of intense fires), intensification of livestock grazing, invasive predators (and meso-predator release via control of the dingo *Canis dingo*—an apex native predator), other invasive animals (e.g. cane toad *Rhinella marinus*) and disease [7, 9, 22–26]. In addition, McKenzie et al. [27] reported that the proportional loss of mammal species was correlated with two predictors: mean annual rainfall (an index of ecosystem productivity), and a composite index of invasive species occurrence, land use, and grazing patterns associated with post-European disturbance. The most persuasive evidence at present points to an interaction between the removal of ground cover via fire and/or grazing (and reduction of shelter and denning sites) and predation, because habitat change exposes small mammals to predators, especially the feral cat (*Felis catus*) [24, 28–30]. A recent review and comparative analysis of ecological and life history traits of declining and non-declining marsupials indicated that, in northern Australia (which is fox-free), predation by feral cats, exacerbated by reduced ground-level vegetation in non-rainforest habitats, is the most likely cause of recent marsupial declines [10]. Experimental testing of these hypotheses suggests that these interactive effects are indeed significant [29, 31].

This paper is a parallel study to our recent analysis of marsupial declines [10], and we ask: (1) what traits are associated with the likelihood of decline of Australian rodents, and are these similar to traits associated with decline in marsupials?; and (2) based on their traits, are rodent species in northern and southern Australia likely to be affected by the same drivers of decline? We treated rodents and marsupials separately in complementary analyses for the following reasons: some life history data and reproductive strategies are very different in the two groups [32]; some ecological traits are restricted to rodents (e.g. being aquatic); the focus on rodents means the results can be presented in a global context, given the world-wide distribution of rodents; and the body size distribution of rodents is much smaller. The interpretation of patterns in marsupials and rodents may therefore differ [32]. Because some threatening processes might act on absolute body size and some on relative body size, separate analysis of rodents and marsupials is predicted to lead to different conclusions to combined models [33]. The identification of common mechanisms of decline across taxa and between northern and southern Australia, and significant differences in the traits of declining families of mammals, will facilitate more cost-effective conservation and management of native mammals across Australia [34].

## Methods

### Data and definitions

From the literature [35–37] we compiled a database of ecological and life history traits (Table 1; S1 File) of all extant Australian rodents including those that may have experienced declines in the modern era due to European influence. For each species we calculated the latitude of the current range centroid, and mean rainfall in the geographic range (modelled using ArcMap 10, with precipitation based on 30-year climate averages and splined using Anuclim 5.2 -http://fennerschool.anu.edu.au/research/products/anuclim - and a 1-km DEM), based on location records of each species in the following databases: The CSIRO Australian National Wildlife Collection, Museum of Victoria, Atlas of NSW Wildlife, NT fauna database, QWILDNET, Biological Survey of South Australia, South Australian Museum, Victorian Biodiversity Atlas Fauna Records, Western Australian Museum specimens database, and the Western Australian DEC Fauna Survey Database. These records are also available via the Terrestrial Ecosystem Research Network mammal data visualisation portal (http://mammalviz.tern.org.au/).

**Table 1:** Ecological and life-history traits used in analyses of the correlates of rodent declines.

Trait	Description	Measurement unit/Coding
***Range***	*pre-decline* geographic range size [based on digitized maps in 35]	
***Female body mass***		mean (g)
***Litter size***	mean number of offspring per litter	
***Reproductive rate***	number of offspring per adult female per year	
***Age of maturity***	age at first reproduction	months
***Diet***	rank based on increasing protein and energy content	1 = grass/leaves; 2 = seeds, forbs, grass, roots, fungi; 3 = nectar, gum, insects or fruit, leaves, insects; 4 = insects or vertebrates (>50%)
***Habitat number***	number of categories of vegetation structure in which the species occurs, with a maximum of 33	
***Habitat openness***	mean habitat, ranked by height and structural complexity of vegetation [38]	0 = grassland or shrubland; 1 = woodland (e.g. Acacia or open Eucalypt woodland); 2 = both woodland and forest; 3 = forest (e.g. dry or wet sclerophyll); 4 = rainforest—including subtropical, tropical or monsoon rainforest
***Rock dependence***	species association with rocky terrain	0 = not in rock outcrop or gibber habitat; 1 = sometimes occurs in rocky habitat; 2 = dependent on rock outcrops
***Hollow dependence***	extent to which species uses hollows	0 = none; 1 = sometimes uses hollows on ground or in trees; 2 = dependent on tree hollows
***Water dependence***	species association with water and wetland habitats	0 = no water association; 1 = partial use of wetland or riparian habitat; 2 = confined to wetland or riparian habitat
***Habit***	level of arboreality	0 = terrestrial; 1 = terrestrial–high ground cover, runways or tunnels in dense litter or grass cover; nests on ground or in burrow; 2 = partial arboreality- terrestrial foraging, arboreal nesting or *vice versa*; 3 = arboreal

Our aim was to compare traits associated with southern declines with those associated with more recent northern declines in the tropics. Accordingly, we classified rodent species as northern or southern depending on whether their current range centroid was north or south of the Tropic of Capricorn. Southern species by this definition included currently declining rodent species from the central arid region, treated separately from northern species in the tropics that have apparently more recently declined in range (post-1950). The ranges of eight species straddled the Tropic. These were included in both the north and the south, i.e. each population was treated as a taxon with distinct traits such as range decline. This north versus south division also correlates roughly with the distribution of feral cats and large introduced herbivores in the north (especially cattle, donkeys, horses, buffalo), and feral cats, foxes, and a range of small to large introduced herbivores (e.g. rabbits, sheep, cattle, camels) in the south [4], which are implicated as possible mechanisms of decline through predation or removal of ground cover [4, 10]. The response variable, proportional range decline, was based on digitized maps of original and current ranges in Van Dyck and Strahan [35] (see S1 File). These data were based on collection records and recent sub-fossils. We included all 61 species of native Australian rodents distributed on the mainland. We did not include the extinct Christmas Island endemic *Rattus nativitatis*, *R*. *macleari*, or the presumed-extinct Bramble Cay melomys (*Melomys rubicola*). We also omitted the false water mouse (*Xeromys myoides*), for which we had no data on status.

### Statistical analysis

We used two modelling approaches: (1) Bayesian mixed-effects beta regression to test whether different traits predicted range declines in the north (tropics) and south (non-tropics) of Australia, while accounting for the effect of phylogeny; and (2) random forest models to test for the strength of association of the traits identified in the multiple beta regression with the dichotomous variable—range decline occurrence (whether the species declined at all or not) [39].

1. The following predictor variables were included in the Bayesian mixed-effects beta regression model: region (factor, 2 levels: north, south), geographic range size (continuous, log-transformed), mean rainfall (continuous, log-transformed), mean female mass (log-transformed), habitat openness (ranked), and mean litter size (continuous, log-transformed). All continuous variables were scaled to have zero mean and unit variance, to aid in numeric model-fitting. Priors for all predictor coefficients were normal with zero mean and standard deviation 1000. We included species as a random effect, with a multivariate normal prior with zero mean and covariance matrix equal to the phylogenetic variance-covariance matrix derived from the phylogeny. We used a rodent phylogeny derived from Cardillo et al. [40], using Ford [41] for resolution of *Pseudomys* and Geffen et al. [42] for *Rattus*. We measured and adjusted for phylogenetic signal using Pagel's λ transformation of the phylogenetic covariance matrix [43]. A Uniform (0, 1.2) prior distribution was used for λ.

The Beta distribution is usually parameterised with shape parameters α and β. We modelled the mean of the Beta distribution for decline using the following re-parameterisation: α_i_ = μ_i_γ and β_i_ = (1−μ_i_)γ, where α_i_ and β_i_ are the shape parameters for the ith species, μ_i_ is the mean for the ith species and γ is related to the dispersion of the distribution. μ_i_ was modelled as a linear function of the predictor variables and species effects, using a logit link function. For γ, we used a Gamma prior distribution with shape and scale parameters = 0.001.

We fitted two models. The first allowed 2-way interactions of all predictor variables with region, to establish whether there was a north-south difference in declines in response to each predictor variable (e.g. if rodents that declined in the north were smaller than those that declined in the south). This model had a large number of parameters, so to test if declines were associated with predictor variables without accounting for north-south differences we also fitted a second simpler main effects model with fewer parameters (increased power), with region and the other predictors included, but without interaction terms.

Models were fitted using Stan version 2.1.0 [44]. Stan implements Bayesian inference using a variant of the Hamiltonian Monte Carlo algorithm [45]. For each model, we ran four chains, each of 1000 iterations for the adaptation phase (discarded), followed by a further 50000 iterations, with no thinning. Post-processing of the chains was performed using the RStan and coda packages for R [44, 46, 47]. We checked for convergence by eye, and by using the Gelman-Rubin diagnostic test [48]. We examined autocorrelation plots to check for lack of independence among iterations, which was minimal. We therefore combined the chains and based our inference on this single chain of length 200000. In the model with interactions, effective sample size for parameter estimates ranged from 5806 (intercept) to 22036 (interaction between region and log female mass). For the simpler main-effects model, effective sample sizes ranged from 6650 (intercept) to 22621 (log rainfall).

2. To test if traits of species that declined differed from those that did not, and to visualise thresholds [39], we also constructed a random forest regression tree for northern and southern Australian rodent species together. This method builds a classification tree by repeatedly splitting the data based on whether they fall above or below a threshold value of each explanatory variable in the model [49]. Because this method identifies interactions in which the same variable repeatedly enters a model at different levels, it can find threshold values (including if there are both upper and lower thresholds) [49, 50].

The relative strength of association of covariates with the response variable can be difficult to interpret, because small changes in values of the covariates can alter their order in the tree [49]. To minimize this possibility and improve classification accuracy, our random forest approach combined a large number of regression trees and evaluated the results by a cross-validation process [39]. Error is reported as an out-of-sample prediction error rate, in which prediction accuracy is determined on a subset of the data different from that used to generate the prediction. We used the package ‘randomForest’ in R [51]. To visualize the results of this analysis, we present a conditional inference tree based on the variables identified as the most strongly associated with the response variable by the random forest analysis. The tree was constructed using the function ‘ctree’ in the R package ‘Party’ [52].

## Results

Nine rodent species have experienced range decline in the northern tropics, comprising 28% of all northern species, while most southern species (n = 31 species, 79.5%) have experienced declines in their range (Table 2). Mean female body mass in southern rodents was half that of northern species (south: 70.9±11.3 g, n = 32 species; north: 141.1±34.5 g, n = 29 species; mean±SE).

**Table 2:** Summary of the number of rodent species that have declined by region.

	Decline category				
	None	Low	Moderate	High	Total
**Northern region**	23	1		8	32
**Southern region**	8	8	2	21	39

Cell values are number of species. Decline categories refer to percent range decline as follows: Low = < 25% decline in range; Moderate = 26–50% decline in range; High = >50% decline in range.

### Beta multiple regression models

**Fig 1:**
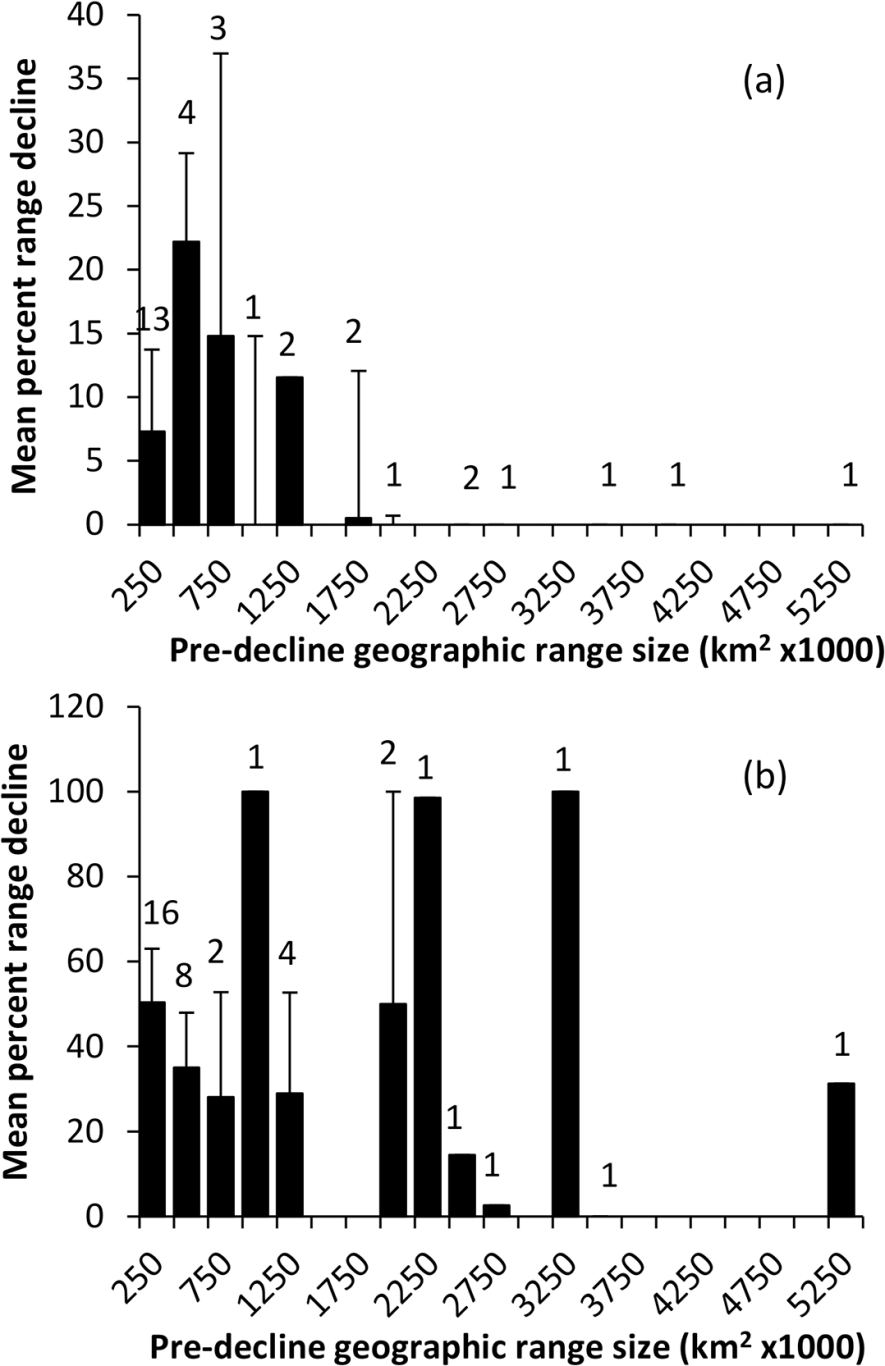
The relationship between pre-decline geographic range and proportional range decline, showing that rodent species with more restricted distributions have declined in both (a) the northern, and especially (b) the southern region. Numbers above bars represent number of species in that category.

**Fig 2:**
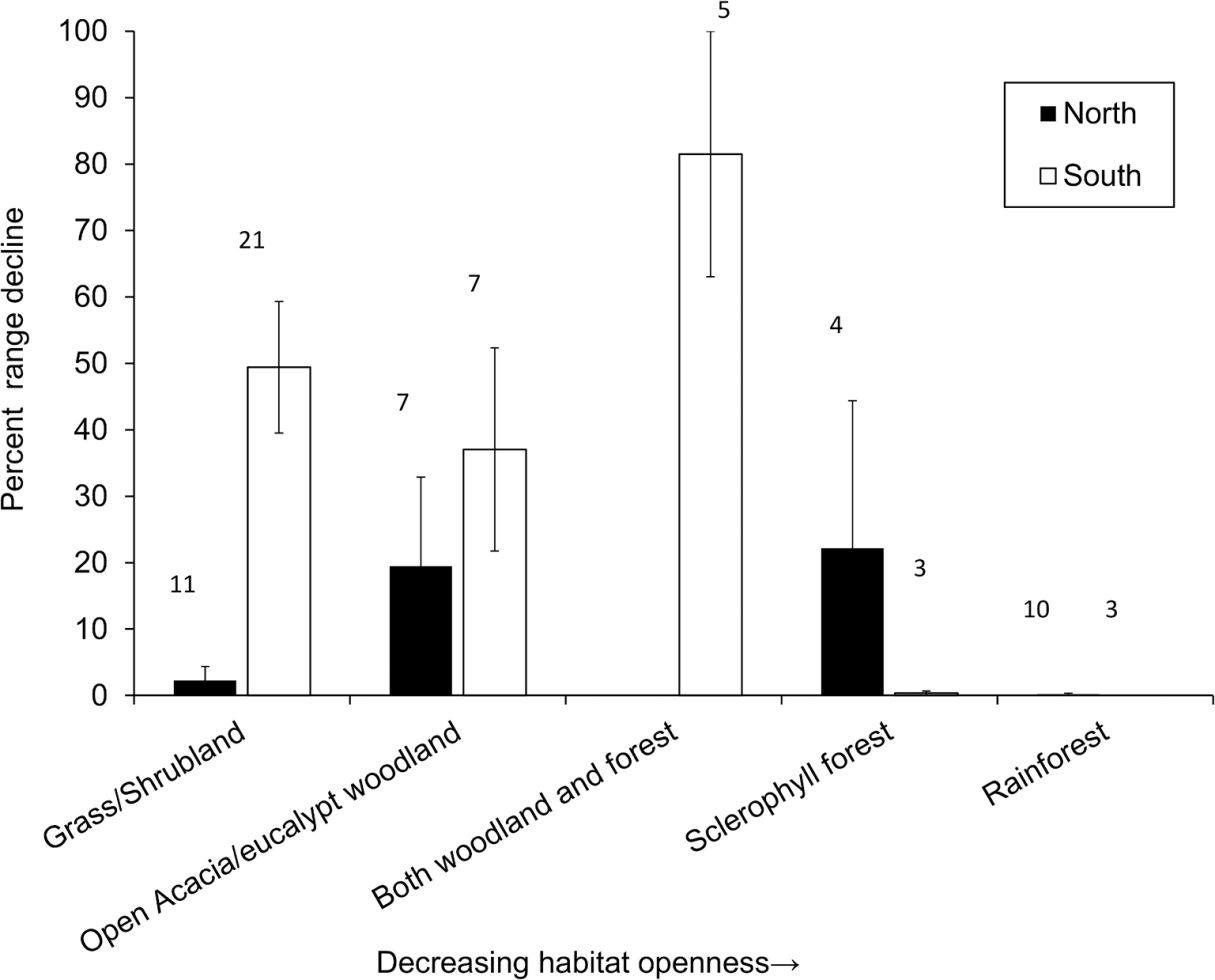
The relationship between habitat openness (ordinal factor) and proportional range decline, showing that rodent species in the more sparsely vegetated habitats declined more, particularly in the southern regions. Numbers above bars represent number of species in that category.

Rodents in southern Australia have undergone significantly greater proportional range decline than species in northern tropical Australia (Table 3). The full model with interactions failed to show that any species traits varied with proportional range decline in different ways in the north and south: there were no significant interactions between region (north and south) and any of the predictor variables (pre-decline geographic range size, rainfall, female body mass, habitat openness, and litter size) (Table 3). We ran this model with fewer parameters to test if proportional decline varied with region as a main effect, and without testing for interactions between each independent variable and region (north vs. south). Greater decline in the south than in the north was evident, pre-decline geographic range was negatively associated with range decline (species with more restricted distributions declined more) (Fig 1) and habitat openness was also negatively associated with range decline so that species in the more sparsely vegetated habitats declined more (Table 4; Fig 2). These sparse vegetation types included semi-arid and arid grasslands and shrublands, open eucalypt woodland and sclerophyll forest in the south, as well as tropical savannas in the north.

**Table 3:** Results of a Bayesian mixed-effects Beta regression model testing for predictors of decline in range (proportional decline) and interactions between region ‘NS’ (presence in northern and tropical Australia versus southern and temperate) and other explanatory variables, using 61 species of rodents.

Predictor	Mean	SE	95% HPDI
Intercept*	-1.9278	0.0073	(-3.0295, -0.8474)
NS*	1.4763	0.0043	(0.5833, 2.3842)
log Range	-0.0844	0.0033	(-0.8174, 0.6631)
log Female mass	0.2257	0.0022	(-0.3491, 0.7975)
log Rainfall	0.1587	0.004	(-0.7052, 1.0541)
Habitat openness	-0.4014	0.003	(-1.149, 0.3441)
log Litter	0.0879	0.0038	(-0.5523, 0.7118)
NS:log Range	-0.5033	0.004	(-1.4359, 0.4206)
NS:log Female mass	0.0143	0.0026	(-0.7336, 0.7713)
NS:log Rainfall	-0.3819	0.0062	(-1.6786, 0.9104)
NS:Habitat openness	-0.2994	0.0047	(-1.4644, 0.8712)
NS:log Litter	-0.1805	0.003	(-0.9342, 0.5689)
γ *	0.5216	0.0007	(0.3593, 0.7437)
λ *	0.7919	0.0024	(0.2369, 1.0053)

95% HPDI is the 95% Highest Posterior Density Interval, and * indicates notable effects (those in which the 95% HPD Interval does not include zero). Gamma (γ) shows the dispersion of the beta distribution, and lambda (λ) designates Pagel’s lambda, a measure of phylogenetic signal.

**Table 4:** Results of a Bayesian mixed-effects Beta regression model testing for predictors of decline in range (proportional decline–see text for explanation), using 61 species of rodents.

Predictor	Mean	SE	95% HPDI
Intercept*	-1.6596	0.0061	(-2.6421, -0.6925)
NS*	1.2177	0.0028	(0.428, 2.0196)
log Range*	-0.4535	0.0019	(-0.8957, -0.0122)
log female mass	0.284	0.0019	(-0.1844, 0.7568)
log Rainfall	-0.1891	0.0018	(-0.7369, 0.3602)
Habitat openness*	-0.6127	0.0025	(-1.1718, -0.0605)
log Litter	-0.0102	0.0024	(-0.5614, 0.5316)
γ *	0.5047	0.0007	(0.3466, 0.7096)
λ *	0.808	0.002	(0.281, 1.0047)

95% HPDI is the 95% Highest Posterior Density Interval, and * indicates notable effects (those in which the 95% HPD Interval does not includes zero). Gamma (γ) shows the dispersion of the beta distribution, and lambda (λ) designates Pagel’s lambda, a measure of phylogenetic signal.

**Fig 3:**
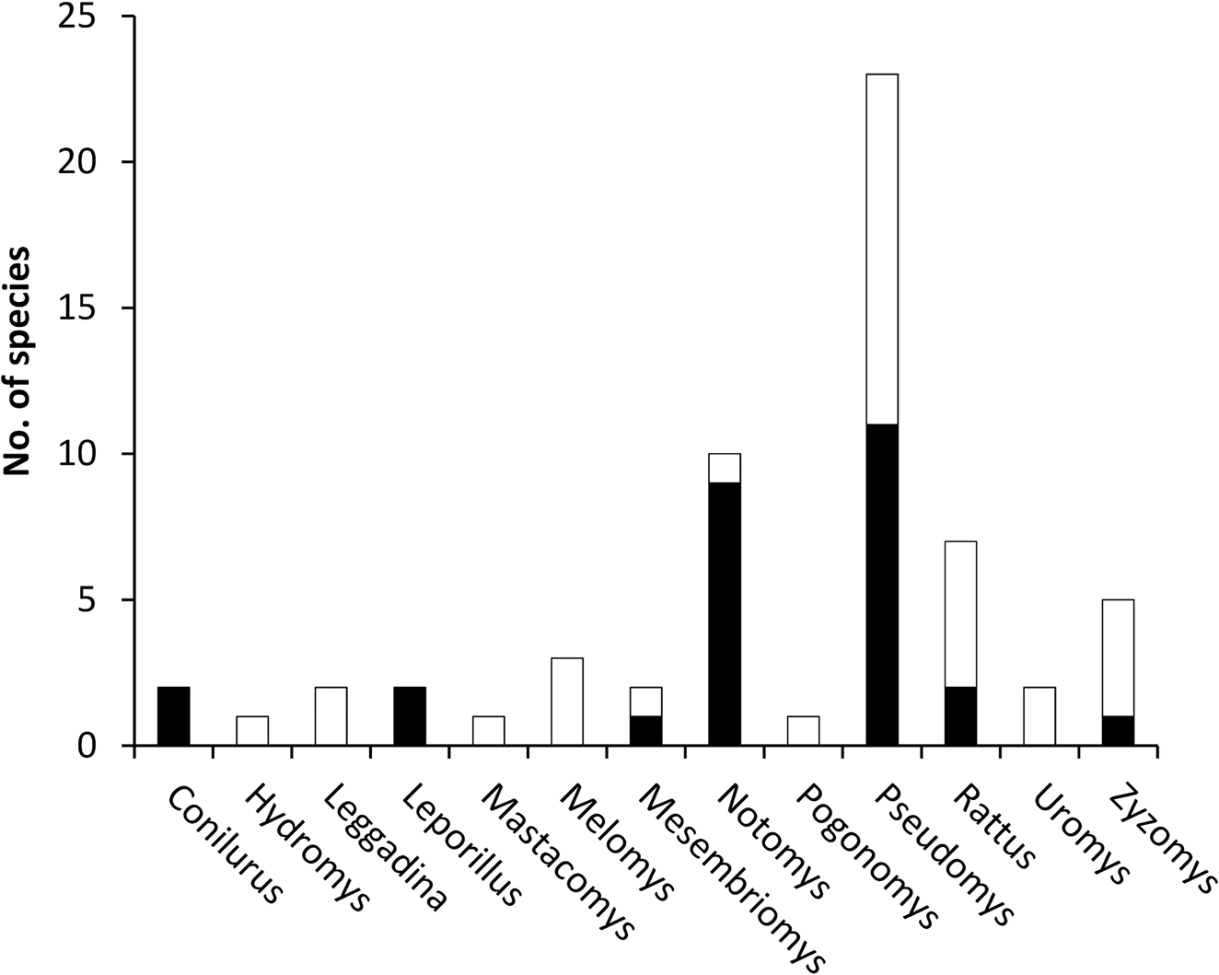
Incidence of species declines within Australian rodent genera. Black bars indicate declining species while white bars indicate species whose range size is stable.

There was considerable variance due to phylogeny: the mean for Pagel's λ for the model with interactions was 0.79 (95% HPDI: 0.35–1.01; Table 3), and for the main-effects model it was 0.81 (0.40–1.01; Table 4). Both 95% HPD intervals contain 1; this demonstrates that a model of Brownian motion for the logit of the decline data (conditional on the given phylogeny) cannot be rejected. More closely related species, for example the many declining species in the speciose genera *Notomys* (hopping mice) and *Pseudomys* (morphologically generalist small native mice) (Fig 3), had similar patterns of decline in the north and south, and this similarity in decline is well-described by the phylogeny, and a Brownian motion model of evolution.

### Random Forest model

**Fig 4:**
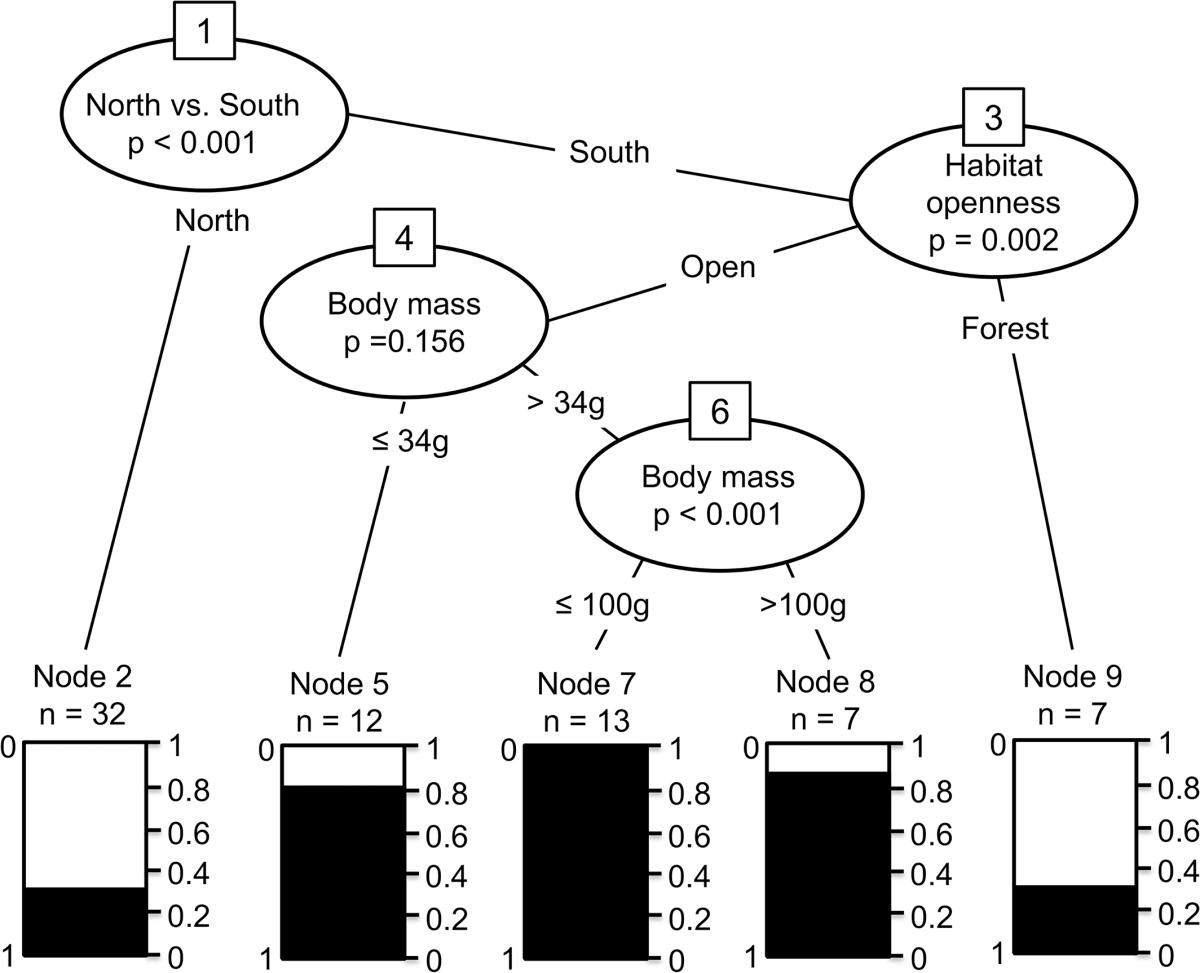
Conditional inference tree based on the variables most strongly associated with range decline from a random forest model. Shading represents the proportion of species that have declined, and *n* is the number of species in each of the final groups. Numbers in boxes represent the node number at which each split occurred. Overall out-of-sample prediction error rate (overall misclassification rate) was 21%. Species at nodes 5, 7 and 8 have all declined greatly (>80% of species). Substantially more species at nodes 5, 7 and 8 (species of all body masses in non-forest vegetation types) have declined than at node 9 (species of all body masses in rainforest and forest vegetation types).

In agreement with the multiple regression approach, our regression tree analysis showed that region was the most important predictor of whether or not rodents declined in range (the first split, Fig 4): substantially more southern rodents have declined in range than northern rodents. The model identified no further traits associated with variation in range decline in northern rodents. The most important variable in the south was habitat structure (Fig 4). The likelihood of any range decline was much greater in open grassland and woodland habitats than in denser forest and rainforest. A third of southern rodents in forest and rainforest categories have declined, but more than 80% of species in grassland and woodland. The model identified no further traits associated with variation in range decline in southern forest rodents, but body mass influenced vulnerability in southern non-forest rodents. All southern rodents of open habitats that have females between 34 g and 100 g have declined in range (node 7, Fig 4), and this proportion is significantly greater than in larger species.

## Discussion

Many species of Australian rodents have experienced severe declines in abundance and distribution in the last 150 years, particularly in southern Australia, but more recently also in northern Australia [3]. We found that phylogeny, latitude and habitat structure differentiated rodents that have undergone substantial range declines from those that have not. Severity of range decline in Australian rodents is strongly skewed according to phylogeny: the genera *Notomys*, *Leporillus* and *Conilurus* (all relatively specialised ‘old endemic’ rodents) have declined most. These declines have been much worse in the south, where large species are now extinct and the species in the more sparsely vegetated non-rainforest habitats have declined more. Regression trees revealed that within this vulnerable subgroup of southern rodents in open habitats (in which a large proportion of all body sizes have declined), currently the greatest declines have occurred in moderately small-bodied species (34–100 g). Our results confirm previous conclusions by affirming the qualitative findings of McKenzie et al. [27], Dickman et al. [53] and Smith and Quin [4]. McKenzie et al. [27] found a strong correlation between both ecosystem productivity (mean annual rainfall) and environmental change caused by post-European disturbance—which both influence habitat structure, and mammal declines in Australia. Dickman et al. [53] postulated that cats caused early declines of small native rodents in open habitats. Smith and Quin [4] found that declines of conilurine rodents were more severe in open habitats (arid centre and temperate woodlands) or habitats that had been modified by grazing or frequent burning, and that cat abundance (based on expert elicitation [54]) was the best predictor of declines among small conilurines (<35 g).

### Vegetation structure

A similar association between sparse vegetation structure and range decline, and conversely a protective effect of dense forest, especially rainforest, has been repeatedly found for marsupials in southern Australia [10, 23, 27, 53, 55], and recently also in the tropics [10]. Our beta multiple regression analyses showed a consistent association between habitat openness and declines, with an overall correlation between sparsely vegetated habitat and declines across Australia, and no evidence that the effect of habitat structure differed between northern and southern Australia. The random forests model did not find significant associations between species traits and decline in northern Australia, but this may be due to low power to detect separate trends in the tropics, where fewer species have declined.

Many authors [4, 10, 23, 53, 56] have argued that fire and grazing are the major landscape-level drivers of environmental change in Australia. Fire and grazing pressure are implicated in the reduction of marsupial and rodent populations via the removal of ground cover and simplification of understorey vegetation [57–59]. The relative influence of the drivers of vegetation simplification such as fire and grazing by livestock (domestic and feral), rabbits (in times past) and irrupting kangaroos, vary according to differences in vegetation type, climate and land use and are geographically variable [4, 27, 60]. Nevertheless, the effect of vegetation simplification is the creation of more suitable open habitat for invasive predators and exposes native mammals to increased predation pressure [61, 62].

The pervasive effect of fire on mammals in northern Australia may be greater than that of grazing [56], though the two factors interact [59]. Recent research has demonstrated that fire extent, a combined index of fire size and frequency, is associated with decline of small mammals in Kakadu National Park, in the Northern Territory of Australia [31]. Impacts of fire and grazing on small mammals can be more severe in open habitats [27, 56, 63]. In such habitats, extensive and frequent fires can result in declines and even extirpation of many small vertebrates, including small mammals [28, 64, 65].

In their landmark review of the causes of decline of the Australian conilurine rodents, Smith and Quin [4] argued that declines in range size were most severe in open habitats, such as are found in the arid centre and temperate woodlands, where cover is reduced by fire and grazing and the rodents are more vulnerable to predation by red foxes and feral cats. However, it is notable that even though fire is infrequent and has little influence in some open habitats, such as the extensive stony deserts, chenopod shrublands, grassy non-spinifex deserts and semiarid shrublands of southern Australia, rodent declines have been marked in these ecosystems [58, 66]. In these environments, overgrazing by livestock and kangaroos, and predation by red foxes and feral cats has been linked to rodent declines [26, 58]. Overall, the mechanism of small mammal decline in open habitats that is associated with fire and with grazing pressure (domestic and feral livestock; rabbits; irrupting kangaroo populations), appears to be the indirect effects of the removal and simplification of ground cover vegetation on survival and reproductive output (from increased predation), and the temporary loss of resources (e.g. food, nesting), rather than direct fire-related mortality [30, 61, 63, 67, 68]. For example, Letnic and Dickman [69] demonstrated, using a longitudinal dataset, that irruptions of rodents in the Simpson desert of central Australia were associated with the La Niña high rainfall phase of the El Niño Southern Oscillation. Because fuel loads built up after La Niña phases they were also associated with extensive wildfires, and these were in turn associated with marked increases in the populations of cats and foxes and ‘hyper-predation’ on rodents [69].

Our finding that habitat structure, particularly vegetation openness, is a key predictor of rodent declines, is very similar to the finding by McKenzie, Burbidge [27] that mammal declines are negatively correlated with both ecosystem productivity (mean annual rainfall) and environmental change caused by post-European disturbance. However, the predation hypothesis (strongly influenced by habitat openness) posed here may be a more plausible explanation for mammal declines than the ‘ecosystem productivity’ hypothesis because: (1) If ecosystem productivity was a pervasive cause of mammals declines then terrestrial and volant mammals should be equally affected. However, bats do not show the same pattern of declines as terrestrial mammals [27, 53], suggesting that something other than, or in addition to, ecosystem productivity is causing declines among terrestrial mammals; (2) The ecosystem productivity hypothesis is couched in terms of habitat degradation (e.g. over-grazing by rabbits) that has bigger impacts in ecosystems of low productivity, which may account for greater declines of some threatened species in low-productivity areas [27]. However, in north Australia, declines have occurred also in habitats that have not been noticeably degraded e.g. tropical savannas inside large National Parks [6], suggesting that declines are caused by a mechanism (i.e. predation by cats) other than ecosystem productivity; (3) Rodent body size-decline relationships are consistent with the critical weight-range of marsupial and rodent prey preferred by foxes and cats, implying predation [70] rather than productivity effects; (4) Lastly, the northern critical weight-range (CWR) of declining mammals is consistent with the southern Australian CWR [70], again supporting predation effects rather than productivity effects.

### Distribution and body size

Our analysis of proportional range decline indicated that rodents with larger pre-decline geographic distributions declined less. This is consistent with global patterns of extinction risk and with ecological theory [71]. Small geographic range size and large body size are the most important global predictors of extinction risk in mammals [50, 72, 73]. Species with small geographic range sizes are more likely to be ecologically specialized, which increases extinction proneness by conferring vulnerability to large scale habitat disturbance and loss [74]. In a qualitative review, Cole and Woinarski [75] also found that arid zone rodents of the Northern Territory of Australia were more likely to have declined if they had smaller geographic ranges. Cross-species analysis of marsupials has not found significant effects of range size on decline [76].

Previous studies concluded that rodent species with larger body mass were more likely to be in decline in arid ecosystems of the Northern Territory of Australia [75] and in general [4]. In contrast to these studies, and to our own earlier study of marsupials [10], we found that body mass was not a major predictor of decline in rodents. Body mass did not predict the severity of range decline in tropical rodents, and was not associated with proportional decline across Australia. Body mass was associated with decline of southern rodents of open habitats: our Random Forest model indicated that moderately small rodents in these vegetation types have declined the most. All 13 southern Australian species of habitat rank less than 2 (more open than forest), heavier than 34 g, but lighter than 100 g, have declined to some extent (Fig 2).

Johnson and Isaac [55] also showed that marsupial declines in arid regions (i.e. grassland and woodland) show a humped relationship with body mass, but this is not the case in mesic regions (typically forest). We found that a very high proportion of rodent species of all body sizes in open habitat (> 80%) have also declined. The association between medium body mass and decline was weaker than the association between habitat and decline. All rodents of open habitats in both northern and southern Australia are smaller (94.9 ± 21.3 g; mean ± SE; n = 50 species) than the size at which marsupials are most vulnerable to declines (>100 g) [55] and within the size range (<220 g) that is preferred by feral cats [7, 77].

Predation by the feral cat and the red fox is thought to be a primary cause of declines of small marsupials in Australia [10]. Our analyses are consistent with predation being an important cause of rodent declines. Both this study and that of Fisher et al. [10] support the hypothesis that an interaction of fire, grazing and predation is the main cause of mammal declines, through the mechanism of reduction of ground cover causing increased risk of predation by both foxes and cats in southern Australia [26, 78] and by cats in northern Australia. There is a long, documented history of the devastating effects of red foxes on small mammal declines in southern Australia and the arid centre [79, 80]. Foxes prey on larger medium-sized mammals (0.5–6.9 kg) [81] than cats. In southern Australia the range of occupancy of foxes and cats overlaps [4]. It is thus possible that that larger-sized rodent species have gone extinct in southern Australia because of the combined impact of cats and foxes. Recently, Colman et al. [57] have linked declines in rodent abundance in temperate forest ecosystems in southern Australia to both increases in macropod grazing pressure/simplification of understorey vegetation and fox abundance following dingo control. However, the role of predation by cats on mammal declines in northern Australia has been less clear (but see [29, 30]).

## Conclusions

The same mechanisms of decline appear to be operating in marsupials and rodents in northern and southern Australia, and habitat structure is an important determinant of declines in both groups. Our results have broad implications for the management of small mammals. First, to manage for small mammal diversity, and indeed the diversity of many other animal and plant taxa, large scale fires, intense grazing practices on either sheep or cattle properties, and the densities of feral herbivores, should be limited to prevent loss of ground cover, coarse woody debris and habitat heterogeneity [82]. Second, threshold levels of ground cover required to maintain biodiversity in different ecosystems need to be further researched, as do the interactions of fire and grazing with these levels. Third, efforts to control feral cats and the red fox must continue and should be increased in open habitat ecosystems. Judicious management of dingo populations (see [80]), entailing their return to some of these ecosystems, or at least the cessation of intense baiting and culling campaigns in many of them, is recommended. The creation of predator-free conservation reserves may be prudent while control measures are put into practice. The commonality of the causes of decline across regional Australia should make broad-scale biodiversity conservation programmes more tractable, co-ordinated and cost-effective.

## Supporting Information

S1 File Life-history traits and ecological correlates of Australian rodent species. (XLSX)
